# Early Molecular Biomarkers in an Amyloid-β-Induced Rat Model of Alzheimer’s Disease: Effects of Kelulut Honey

**DOI:** 10.3390/ijms27021059

**Published:** 2026-01-21

**Authors:** Ammara Shaikh, Fairus Ahmad, Jayakumar Murthy, Seong Lin Teoh, Mohamad Fairuz Yahaya

**Affiliations:** 1 Department of Anatomy, Faculty of Medicine, Universiti Kebangsaan Malaysia, Kuala Lumpur 56000, Malaysia; ammarashaikh9@gmail.com (A.S.); fairusahmad@ukm.edu.my (F.A.); teohseonglin@ukm.edu.my (S.L.T.); 2 Department of Physiology, Faculty of Medicine, Universiti Kebangsaan Malaysia, Kuala Lumpur 56000, Malaysia; jayakumar@ukm.edu.my

**Keywords:** Alzheimer’s disease, molecular markers, ELISA, rat model

## Abstract

Alzheimer’s disease (AD) is the leading cause of dementia worldwide, characterized by progressive neurodegeneration and cognitive decline. Early diagnosis remains critical for enabling timely intervention. However, detecting the earliest pathological changes is challenging due to the limited availability of reliable biomarkers that reflect early disease pathology in experimental models. This study evaluated molecular markers associated with AD-related processes in a rat model inoculated with human amyloid β (Aβ)_1-42_ peptides. We assessed the levels of biomarkers: Aβ_1-42_, Aβ_42_, phosphorylated tau, monocyte chemoattractant protein-1 (MCP-1), nuclear factor kappa B (NF-κB p65) and superoxide dismutase 1 (SOD1) in hippocampal tissue and serum using enzyme-linked immunosorbent assay. A treatment group receiving Kelulut honey was included to evaluate biomarker responsiveness. Results showed significant elevation in hippocampal Aβ_1-42_ and phosphorylated tau in diseased rats, with changes in inflammatory markers MCP-1 and NF-κB p65, whereas no significant change was observed in oxidative stress marker SOD1. Serum levels of Aβ_1-42_ and MCP-1 did not differ significantly between groups, indicating limited peripheral sensitivity after a month of disease induction. These findings suggest that amyloid-, tau-, and inflammation-related markers in hippocampal tissue may be informative for early pathological changes in this acute model, while serum markers showed limited sensitivity.

## 1. Introduction

Dementia affects more than 55 million people worldwide, with Alzheimer’s disease (AD) being the leading cause [1]. Despite significant advances in therapeutic research, AD remains without a definitive cure. It is characterized by progressive neurodegeneration, cognitive decline, and distinct neuropathological hallmarks, including amyloid β (Aβ) deposition, tau hyperphosphorylation, oxidative stress and chronic neuroinflammation [2]. Although these features are well established in the advanced stages of AD, detecting the earliest pathological changes remains challenging, thereby limiting opportunities for early diagnosis and intervention.

Recent research has established Aβ_42/40_ and phosphorylated tau at threonine 217 (p-tau217) as the most consistent early biomarkers of AD. Highly sensitive plasma assays now achieve diagnostic accuracy equivalent to cerebrospinal fluid (CSF) measurements, enabling early, non-invasive detection and longitudinal monitoring [3]. Moreover, converging experimental and clinical evidence indicates that soluble Aβ oligomers act as primary neurotoxic species that initiate tau hyperphosphorylation and disrupt synaptic integrity prior to amyloid plaque deposition and the onset of overt neurodegeneration [4,5]. Interpreting Aβ and tau results together therefore provides stronger mechanistic coherence and supports their concurrent evaluation in early experimental and clinical AD models.

In parallel, current genetic and experimental studies have strengthened the role of neuroinflammation as a contributing factor in AD rather than a secondary response. The Monocyte Chemoattractant Protein-1 (MCP-1) and Nuclear Factor Kappa-Light-Chain-Enhancer of Activated B cells (NF-κB) pathways have been implicated as important amplifiers of amyloid accumulation and neuronal stress [6,7,8]. The antioxidant enzyme and oxidative stress marker superoxide dismutase 1 (SOD1) signifies early oxidative imbalance that exacerbates inflammatory signalling, thereby contributing to subsequent neuronal dysfunction [9]. Furthermore, combining blood-based biomarkers (BBMs) such as Aβ_42/40_, p-tau217, neurofilament light chain (NfL), and glial fibrillary acidic protein (GFAP) enhances the predictive accuracy for identifying individuals at risk of developing dementia [10]. These biomarkers support scalable and non-invasive early screening, while hippocampal enzyme-linked immunosorbent assay (ELISA) data can serve as complementary mechanistic validation to strengthen peripheral biomarker interpretation.

The present study aimed to evaluate molecular markers associated with AD-related processes in an acute Aβ_1-42_ injection model. In this study, an AD ratmodel was established, designed primarily as a preclinical biomarker assessment platform rather than a therapeutic efficacy trial. An additional treatment group receiving Kelulut honey was included as a comparative intervention, based on prior evidence indicating its neuroprotective and anti-inflammatory properties in experimental AD and other neurodegenerative diseases, including but not limited to our earlier work [11,12,13,14,15]. The purpose of including this group was to examine whether established AD-related molecular biomarkers could detect consistent and directionally relevant biological changes within an acute Aβ model, rather than to evaluate or claim definitive therapeutic benefit. This approach allows validation of biomarker sensitivity and responsiveness in the presence of a biologically active intervention, thereby strengthening the translational relevance of the selected markers in early-stage AD pathology.

## 2. Results

ELISA analyses were performed on hippocampi homogenates (for amyloid, p-tau, MCP-1, NF-κB p65, SOD1) and serum (for amyloid and MCP-1). The parameters included two disease markers: Aβ and p-tau, SOD1 as an oxidative stress marker, and NF-κB p65 as a neuroinflammatory marker. Additionally, MCP-1 (C-C motif chemokine ligand 2, CCL2), a key mediator of chronic inflammation and AD pathological cascade was also assessed.

### 2.1. Human Amyloid β_1-42_ and Rat Amyloid β_42_ Levels in Brain

**Figure 1:**
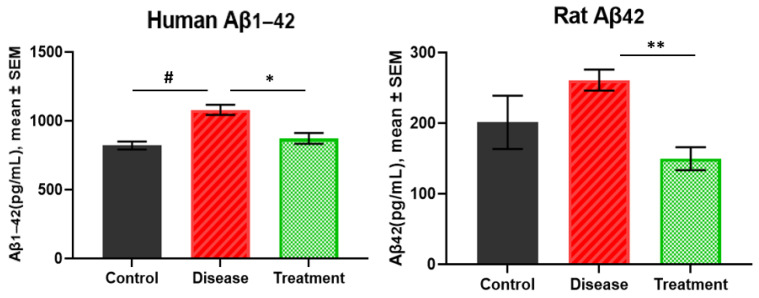
The results of human Aβ_1-42_ and rat β_42_ in rat hippocampus. * *p* < 0.05, ** *p* < 0.01, # *p* < 0.001.

Human Aβ_1-42_ levels were highest in the disease group (1079.76 ± 37.14 pg/mL), followed by the treatment (872.29 ± 39.79 pg/mL) and control groups (820.32 ± 31.43 pg/mL). Levels were significantly higher in the disease group than in the treatment group (*p* = 0.0110). Moreover, the protein levels in the disease group were significantly higher than the control group (*p* = 0.0006). However, no difference was detected between the treatment group and the control group (*p* = 0.5641) (Figure 1).

Next, Figure 1 also shows that rat Aβ_42_ levels were highest in the disease group (260.91 ± 14.90 pg/mL) compared to the treatment (149.69 ± 16.28 pg/mL) and control groups (201.04 ± 37.78 pg/mL). The Aβ_42_ levels were significantly lower in the treatment group compared to the disease group (*p* = 0.0020). However, there were no significant differences between the control group (*p* = 0.4524 and *p* = 0.3420, respectively) for either comparison.

### 2.2. p-tau Levels

**Figure 2:**
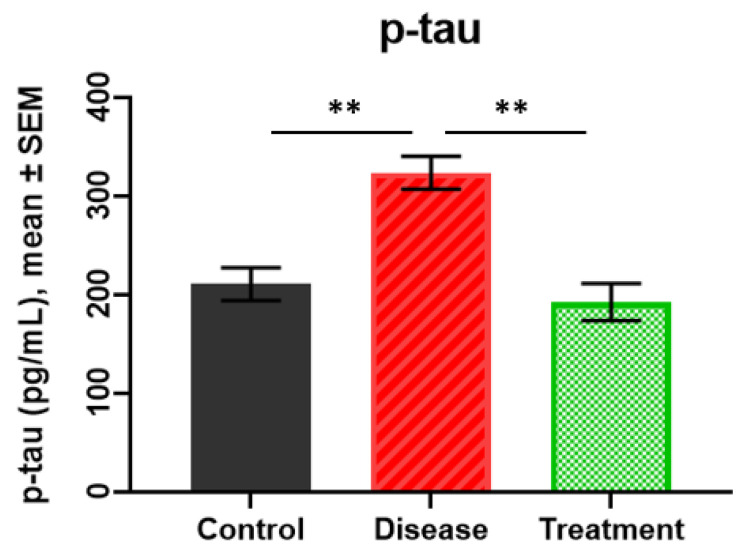
The results of p-tau in rat hippocampus. ** *p* < 0.01.

The lowest p-tau levels were detected in the treatment group (192.563 ± 18.80 pg/mL), followed by the control group (210.77 ± 16.74 pg/mL) and the disease group (323.81 ± 16.80 pg/mL) Statistical analysis revealed a significant increase in the disease group compared to the treatment (*p* = 0.0017) and control groups (*p* = 0.0012). However, the p-tau level in the treatment group was not significantly different from that in the control group (*p* = 0.7559) (Figure 2).

### 2.3. MCP-1 and NF-κB p65 Levels

**Figure 3:**
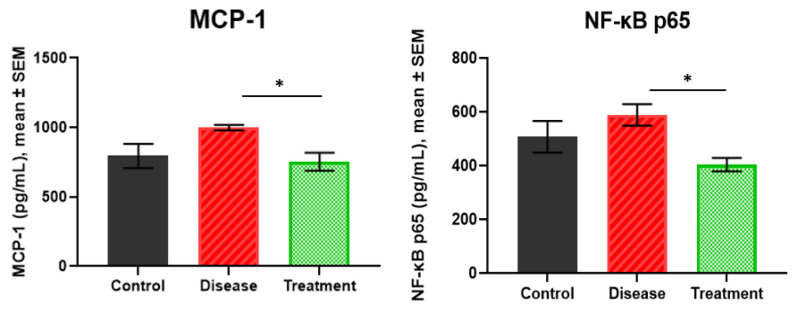
The results of MCP-1 and NF-κB p65 in rat hippocampus. * *p* < 0.05.

The MCP-1 levels were 795.44 ± 86.89 pg/mL in the control group, 998.20 ± 19.43 pg/mL in the disease group, and 753.61 ± 64.81 pg/mL in the treatment group. The treatment group showed a lower MCP-1 concentration than the disease group (*p* = 0.0374). No significant difference was observed when the disease (*p* = 0.1120) and treatment (*p* = 0.9217) groups were compared to the control group (Figure 3).

The NF-κB p65 levels were highest in the disease group (588.50 ± 39.99 pg/mL), followed by the control (507.18 ± 58.60 pg/mL) and treatment groups (403.44 ± 24.92 pg/mL). The disease group had significantly higher levels than the treatment group reflecting AD-related neuroinflammation and its attenuation following treatment (*p* = 0.0106). However, the control group was not significantly different from the disease (*p* = 0.5045) and treatment groups (*p* = 0.2768) (Figure 3).

### 2.4. SOD1 Levels

**Figure 4:**
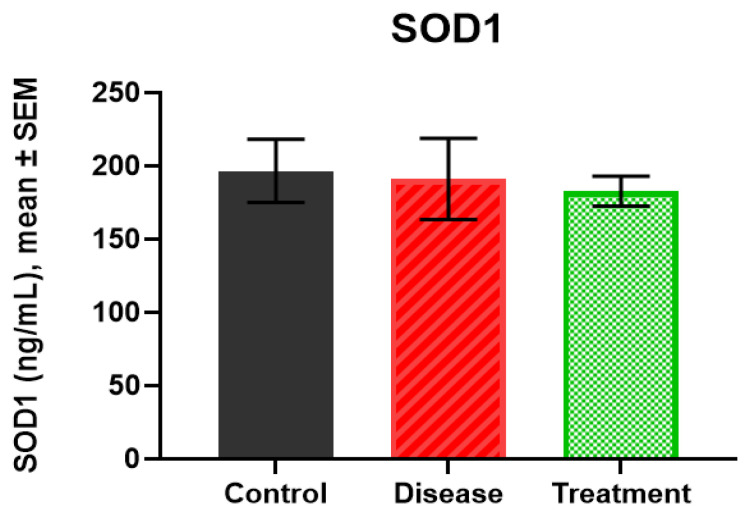
The results of SOD1 levels in rat hippocampus.

SOD1 levels were similar across groups, control group (196.66 ± 21.46 ng/mL), disease (191.16 ± 27.78 ng/mL) and treatment (182.75 ± 10.21 ng/mL). No significant differences were detected between groups (all *p* > 0.05) (Figure 4).

### 2.5. Serum Levels of Aβ_1-42_ and MCP-1

**Figure 5:**
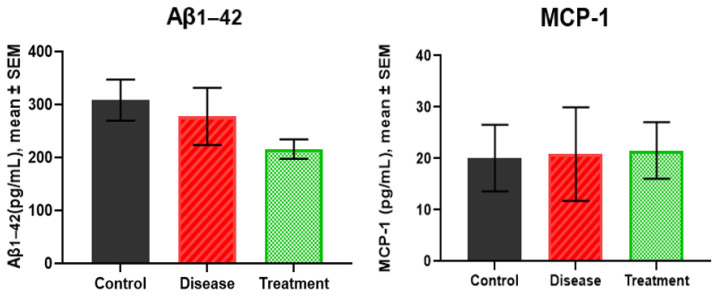
The results of Aβ_1-42_ and MCP-1 in rat serum.

Serum was analyzed for the levels of Aβ_1-42_ and MCP-1 to observe any significant results of these markers for early detection of AD in disease-induced rodents (Figure 5). Statistical analysis showed non-significant *p*-values across all group comparisons for both biomarkers.

## 3. Discussion

**Figure 6:**
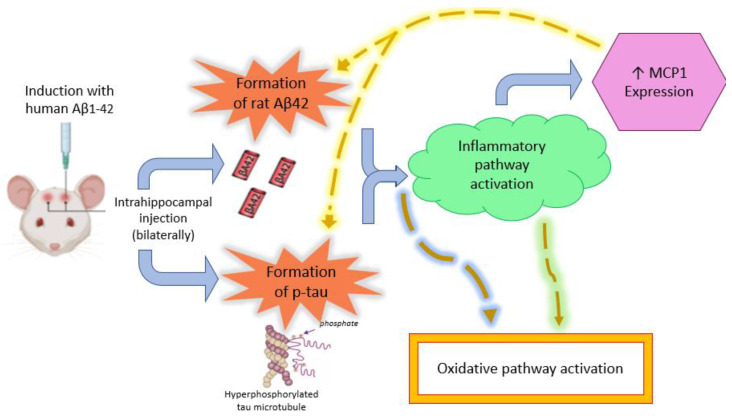
Illustration showing the induction of AD with human Aβ_1-42_ that results in formation of rat Aβ_42_ and the initiation of tau pathology. The two disease markers then likely activate inflammatory pathway which results in increased expression of MCP-1, which in turn can further stimulate amyloid and tau accumulation. The oxidative pathway is later activated by amyloid and tau with/without an indirect influence of the inflammatory pathway. Thick blue arrows indicate the progression of disease, dotted brown arrows represent the likely effect of one pathology on another, and **↑** indicates increase.

This study evaluated molecular markers associated with AD-related processes in an acute Aβ_1-42_ hippocampal injection model and assessed their responsiveness within this early pathological context. The results demonstrate that molecular markers, particularly human Aβ_1-42_ and rat p-tau, showed significant elevations in AD-induced rodents compared to controls. In this acute model, human Aβ_1-42_ showed larger group differences than rat Aβ_42_, likely reflecting the direct administration of human peptides. These markers appeared responsive to the induced pathology, though no correlation analyses were performed. Figure 6 shows the induction of AD with human Aβ_1-42_ that is thought to result in the initiation of tau pathology and the formation of rat Aβ_42_.

It is important to note that post-translational modifications, including the presence of Aβ isomers, may occur in the brain during incubation and could potentially contribute to or modulate AD induction. However, such modifications were not assessed prior to the intrahippocampal injection in the present study. Similarly, additional Aβ isoforms, such as Aβ_1-40_ or other isomers, may form in the AD-induced rat brains and could influence the progression of pathology and the levels of other central markers. Nonetheless, the focus of this study was on the specific Aβ_1-42_ isomer used for induction, and only this form was measured post-injection. Future studies should consider the potential contributions of other Aβ isoforms and post-translational modifications to provide a more comprehensive assessment of AD-related molecular changes [16].

The presence of Aβ_42_ and Aβ_1-42_ in the control group is likely due to the physiological production of these peptides. This finding is also consistent with the similarity between human and rodent Aβ sequences [17]. NF-κB p65 and MCP-1 showed patterns consistent with inflammatory activation, although not all comparisons reached significance. The lack of significance between control and disease groups for NF-κB p65 may reflect variability or mixed central/peripheral contributions [18,19]. The correlation between elevated NF-κB p65 and MCP-1 levels further supports their functional link through the MCP-1/NF-κB pathway [20,21]. Previous studies have also reported increased MCP-1 expression in AD, which can in turn promote amyloid and tau pathology [22,23,24].

Although reduced SOD1 levels have been reported in AD and increased SOD1 activity is generally associated with enhanced antioxidant defence and improved disease prognosis [9,25], SOD1 levels did not differ significantly between groups in the present study. This suggests that oxidative alterations may not yet be detectable at this acute time point or that additional oxidative stress markers are required for a more sensitive and comprehensive assessment. The upward trends in NF-κB p65 and MCP-1 may reflect early inflammatory activity. This pattern is consistent with findings from acute Aβ-induced rodent models, but should be interpreted cautiously [26,27]. These results highlight the need for future research using broader oxidative marker panels to better capture redox dynamics during the early phases of AD.

Although hippocampal markers showed measurable changes, human Aβ_1-42_ and MCP-1 did not differ significantly in serum, reflecting limited peripheral sensitivity in this acute model. This suggests that peripheral biomarkers may be less informative at early time points in this model.

Together, the findings show that amyloid, p-tau, and inflammation-related markers respond to the acute Aβ_1-42_ insult, though interpretation should consider the single time point and acute nature of the model. AD neuropathology and inflammatory changes were detectable, though their temporal sequence cannot be confirmed. However, a hypothesized sequence of events is mentioned in Figure 6. Unlike in humans, where oxidative damage may precede amyloid formation, the present rodent model involves the direct injection of toxic amyloid, which likely triggers the inflammatory pathway first, followed by oxidative changes. Furthermore, the injection of human amyloid peptides seems to stimulate the production of endogenous rat amyloid analogues [28]. The observed p-tau pathology may thus develop directly in response to the neurotoxic effects of the injected human amyloid, as reflected by the highly significant alterations observed.

The observed patterns are compatible with known amyloid–tau–inflammation interactions, (at least in the AD-induced rodent models), but causal or temporal sequencing cannot be established from this cross-sectional study (Figure 6). Soluble Aβ oligomers likely initiate synaptic dysfunction and activate kinases such as GSK-3β and CDK5, leading to tau hyperphosphorylation and neurofibrillary tangle formation [29]. Concurrently, Aβ aggregates engage microglial receptors, including toll-like receptors, triggering the neurotoxic astrocyte phenotype conversion mainly by NF-κB activation and subsequent proinflammatory cytokines and chemokines release [30]. Among these secreted mediators, MCP-1 appears to play a central role by engaging glial cells and initiating a feed-forward inflammatory cascade: MCP-1 recruits and activates microglia and astrocytes, which in turn potentiate NF-κB signalling, thereby further up-regulating MCP-1 expression and sustaining chronic neuroinflammation [31]. This MCP-1 and NF-κB feedback contributes to redox imbalance and mitochondrial dysfunction, thereby exacerbating neuronal injury and amplifying the amyloid–tau–glial cascade [32].

The mechanistic relationships demonstrated through ELISA-based hippocampal analyses in this study provide foundational evidence for understanding how brain-specific biomarker changes translate into peripheral signals measurable in blood. The present findings align with contemporary views that AD pathology extends beyond amyloid accumulation alone, encompassing inflammatory and oxidative pathways that may be reflected differently across tissue and peripheral biomarkers [33]. Such mechanistic validation is essential to interpret and refine current BBM models including plasma Aβ_42/40_, p-tau217, NfL, and GFAP, which have shown predictive accuracy for AD up to 10 years before clinical onset [10,34]. The observed brain-level alterations of Aβ_1-42_ and p-tau in this study parallel these BBM signatures, reinforcing the notion that hippocampal molecular pathology underlies early peripheral biomarker emergence.

Kelulut honey was selected as a comparative intervention based on the neuroprotective effects demonstrated in our previous study [11]. In the present investigation, its inclusion was intended to assess biomarker responsiveness rather than to evaluate therapeutic efficacy. Overall, the findings indicate that amyloid-, p-tau-, and inflammation-related markers represent the most promising candidates for the early detection and potential management of AD.

## 4. Materials and Methods

### 4.1. Animal Model and Experimental Design

A total of 20 male Sprague Dawley rats (~3 months old, ~280–380 g) were supplied by the Laboratory Animal Research Unit, Universiti Kebangsaan Malaysia (UKM). The rats were randomly divided into three groups: Control (*n* = 9), disease group (*n* = 6), and treatment group (*n* = 5).

**Figure 7:**
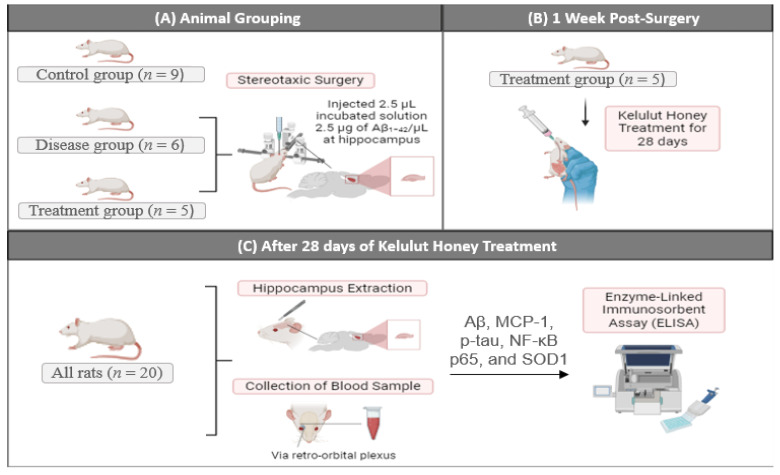
Schematic representation of the experimental design and procedures. (**A**) Animals were divided into control (*n* = 9), disease (*n* = 6), and treatment (*n* = 5) groups. The disease and treatment groups underwent Aβ injection into the hippocampus via stereotactic surgery. (**B**) One-week post-surgery, the treatment group received oral Kelulut honey for 28 days. (**C**) After 28 days, hippocampal tissues and blood samples were collected for ELISA analysis of amyloid, MCP-1, p-tau, NF-κB p65, and SOD1.

Throughout the study, the rats were kept in the Animal Unit, Department of Anatomy, Faculty of Medicine, UKM, in a conventional air-conditioned laboratory environment (25–27 °C, 12 h day–night cycle). Each rat was placed in a separate cage and was provided with rat pellets and tap water ad libitum for seven days as an acclimatization period to adapt to the new place before the commencement of the study. All procedures were carried out in accordance with the institutional guidelines for animal research surgical procedures of Universiti Kebangsaan Malaysia Research and Animal Ethics Committee (UKMAEC) with approval number: ANAT/2022/FAIRUZ/20 JULY/1255-JULY2022-AUGUST-2023. This study builds on our previous study investigating the effects of Kelulut honey on AD [11], with the current focus on evaluating molecular markers sensitive to early pathological progression. All animals survived to the study endpoint, and no animals were excluded. Figure 7 summarizes the experimental design used in this study.

### 4.2. Amyloid β_1-42_ Peptide Incubation

A total of 1 mg human Aβ_1-42_ (Targetmol^®^, Boston, MA, USA) was dissolved in 20 µL dimethyl sulfoxide (Heiltropfen) to make ~21 µL of stock solution, which was divided into 2.5 µL aliquots and stored at −80 °C. For experimental use, one aliquot was diluted at a ratio of 1:19 in distilled water (dH_2_O), which was 2.5 µL stock solution into 47.5 µL of dH_2_O, to prepare 50 µL of working solution. This working solution (125 μg Aβ_1-42_) was incubated at 37 °C for 7 days, protected from light. A dose of 6.25 µg of Aβ_1-42_ was selected to be injected into each hippocampus, consistent with our previous study [11].

### 4.3. Stereotaxic Surgery

The rats from the disease and treatment groups were anesthetized with ketamine-xylazine (1:4 dilution in dH_2_O, 0.5 mL/300 g body weight, intraperitoneally). The head of the anesthetized rat was shaved, sterilized with 75% ethanol, and fixed to the stereotaxic apparatus. Local lidocaine was applied, and an eye cream was used to prevent corneal drying, after which a longitudinal incision was made to expose the cranium. The dorsal hippocampus was localized on both sides in relation to bregma according to the Paxinos and Watson rat brain atlas at AP = −3.8 mm and ML = ±2.2 mm. A burr hole was drilled on both sides. Each hippocampus was injected at DV = −3.4 mm with a volume of 2.5 µL incubated solution containing 2.5 µg of Aβ_1-42_/µL using a 10 µL microinjector (Hamilton^®^, Reno, NV, USA) at a speed of 0.5 µL/min. The microinjector was held at the site for an additional 2–3 min to prevent reflux. Next, the microinjector was removed, after which the wound was stitched with a self-absorbable suture, and an antiseptic cream was applied. In addition, 0.5 mL of 0.9% normal saline was injected subcutaneously to prevent dehydration. Rats were kept on a heating pad to maintain body temperature until recovery and returned to their cages.

### 4.4. Kelulut Honey Treatment

After one week of recovery, rats in the treatment group received Kelulut honey orally at 1 g/kg body weight daily for 28 days, as previously described [11]. The Kelulut honey was diluted in dH_2_O (1:1 ratio) and administered by oral gavage using an 18 G feeding needle, which remained in place for 5 s post-administration to prevent reflux. Rats were observed to ensure the absence of distress, and body weight was monitored every other day.

### 4.5. Blood Collection and Brain Sectioning for ELISA Analysis

At the end of 28-day treatment period, blood samples were collected via the retro-orbital plexus and kept in labelled Eppendorf tubes. The tubes were quickly centrifuged at 5000× *g* for 5 min at 4 °C. The clear supernatant was then removed from the sediment and stored at 80 °C until analysis. On the other hand, the rats were euthanized, and their brains were extracted on a chill plate for ELISA analysis. One of the two cerebral hemispheres was incised to collect the hippocampus which was subsequently stored in a labelled Eppendorf tube. The tube was placed on dry ice and sprayed with 70% ethanol to snap-freeze the sample. The frozen samples were kept at −80 °C until analysis. Prior to homogenization, the hippocampal samples were thawed on ice, and the residual blood was removed by washing the tissue with pre-cooled phosphate-buffered saline (PBS). Samples (0.5 g) were homogenized with PBS (1:9, *w*/*v*; 4.5 mL PBS per 0.5 g hippocampal tissue) using an iron bead homogenizer. Homogenates were centrifuged at 5000× *g* for 5 min at 4 °C. The supernatant was immediately collected and stored at −80 °C until ELISA reading. Commercial ELISA kits were used for detection: human Aβ_1-42_ ELISA kit (ELK Biotechnology, Catalogue no. ELK2100), rat Aβ_42_ (Fine test, Catalogue no. ER0755), rat p-tau ELISA kit (Fine test, Catalogue no. ER1304), rat MCP-1 (ELK Biotechnology, Catalogue no. ELK5504), rat NF-κB p65 ELISA kit (Fine test, Catalogue no. ER1187), and rat superoxide dismutase 1 (SOD1) ELISA kit (Fine test, Catalogue no. ER0332).

### 4.6. Statistical Analysis

Statistical analyses were performed using GraphPad Prism (version 8.0.2). Data are presented as mean ± SEM. Due to unequal and small group sizes, group comparisons were conducted using Brown–Forsythe and Welch’s one-way ANOVA, followed by Games–Howell post-hoc tests for multiple comparisons. This approach was chosen to provide robustness against violations of homogeneity of variance. A *p* value < 0.05 was considered statistically significant. 

## 5. Conclusions and Future Directions

In conclusion, this study demonstrates that hippocampal levels of Aβ and phosphorylated tau increase following acute Aβ_1-42_ injection, reflecting early amyloid- and tau-related pathological changes in this experimental model. These biomarkers showed significant sensitivity in rodent models, supporting their reliability as indicators of AD-related pathology [34]. Inflammatory markers MCP-1 and NF-κB p65 exhibited changes consistent with early neuroinflammatory activation, although not all comparisons reached statistical significance. Collectively, these findings align with emerging evidence suggesting that inflammatory responses may occur at early stages of disease progression, potentially preceding detectable oxidative stress [35]. Importantly, the results underscore the value of hippocampal-specific analyses over peripheral measurements, particularly during the initial phases of pathology.

Future research should focus on expanding the panel of oxidative stress markers to achieve a more comprehensive characterization of redox dynamics in AD. In addition, studies exploring the potential of brain-based biomarkers for early detection and disease monitoring are warranted. Further investigation of neuroinflammatory mechanisms, particularly the MCP-1/NF-κB pathway, may reveal novel therapeutic targets for AD. The translational application of these findings, including the development of brain-based biomarkers for clinical use, warrants further investigation to determine their potential utility in early diagnosis and treatment response monitoring. The Kelulut honey group was included as an experimental contrast to assess biomarker responsiveness based on prior evidence of neuroprotective properties; however, interpretation of these effects is limited, as therapeutic efficacy was not the primary objective of the present study.

Several limitations should be considered when interpreting these findings. First, the study design included a single time point at 35 days (7 days recuperation + 28 days) following Aβ_1-42_ administration, precluding assessment of the temporal sequence or causal progression of amyloid, tau, inflammatory, and oxidative changes. Longitudinal analyses would be necessary to clarify whether inflammatory activation precedes oxidative imbalance or whether these processes evolve concurrently. Second, the intrahippocampal Aβ_1-42_ injection model represents an acute or subacute injury rather than the chronic, progressive pathology characteristic of sporadic human AD. Although this model is well established for inducing rapid amyloid-related changes, it does not fully recapitulate the multifactorial and slowly evolving nature of the disease, and extrapolation to long-term mechanisms should therefore be made with caution. In addition, oxidative stress assessment was limited to a single marker, SOD1, which may not capture the complexity of redox alterations, highlighting the need for inclusion of additional markers such as catalase, glutathione peroxidase, lipid peroxidation products, or protein oxidation in future studies.

Finally, serum biomarkers did not exhibit significant differences among experimental groups. Rather than representing a limitation of the study, this finding highlights the limited sensitivity of peripheral measures at early stages of Aβ-induced pathology and underscores the greater reliability of central molecular markers for early biomarker identification. Accordingly, serum-based findings should be interpreted cautiously in a translational context, particularly with respect to early disease detection.

## Data Availability

The original contributions presented in this study are included in the article. Further inquiries can be directed to the corresponding author.

## Abbreviations

The following abbreviations are used in this manuscript:

AD	Alzheimer’s Disease
MCP-1	Monocyte Chemoattractant Protein-1
NF-κB p65	Nuclear Factor Kappa-Light-Chain-Enhancer of Activated B cells p65 subunit
SOD1	Superoxide Dismutase 1
Aβ	Amyloid Beta
p-tau	Phosphorylated Tau
p-tau217	Phosphorylated Tau at threonine 217
GSK-3β	Glycogen Synthase Kinase 3 Beta
CDK5	Cyclin-Dependent Kinase 5
NfL	Neurofilament Light chain
GFAP	Glial Fibrillary Acidic Protein
CSF	Cerebrospinal Fluid
UKM	Universiti Kebangsaan Malaysia
UKMAEC	Universiti Kebangsaan Malaysia Research and Animal Ethics Committee
dH2O	Distilled Water
ELISA	Enzyme-Linked Immunosorbent Assay
PBS	Phosphate-Buffered Saline
BBM	Blood-Based Biomarker
